# Seroprevalence of *Toxoplasma gondii* Infection in Refugee and Migrant Pregnant Women along the Thailand–Myanmar Border

**DOI:** 10.4269/ajtmh.16-0999

**Published:** 2017-04-17

**Authors:** Bert J. D. van Enter, Yee-Ling Lau, Clare L. Ling, Wanitda Watthanaworawit, Yaowalark Sukthana, Wenn-Chyau Lee, François Nosten, Rose McGready

**Affiliations:** 1 Shoklo Malaria Research Unit, Mahidol-Oxford Tropical Medicine Research Unit, Faculty of Tropical Medicine, Mahidol University, Mae Sot, Thailand;; 2 Department of Parasitology, Faculty of Medicine, University of Malaya, Kuala Lumpur, Malaysia;; 3 Mahidol-Oxford Tropical Medicine Research Unit, Faculty of Tropical Medicine, Mahidol University, Bangkok, Thailand;; 4 Department of Protozoology, Faculty of Tropical Medicine, Mahidol University, Bangkok, Thailand;; 5 Singapore Immunology Network (SIgN), A*STAR, Singapore, Singapore;; 6 Nuffield Department of Medicine, Centre for Tropical Medicine and Global Health, University of Oxford, Oxford, United Kingdom

## Abstract

*Toxoplasma gondii* primary infection in pregnancy is associated with poor obstetric outcomes. This study aimed to determine the seroprevalence of *Toxoplasma* infection in pregnant migrant and refugee women from Myanmar attending antenatal care in Thailand. A random selection of 199 residual blood samples from first antenatal screen in 2014–2015 was tested for *Toxoplasma* IgG and IgM antibodies. Seroprevalence of *Toxoplasma* infection was 31.7% (95% confidence interval = 25.6–38.4). Avidity testing in the three positive IgM cases indicated all were past infections. Multiparity (≥ 3 children) was significantly associated with higher *Toxoplasma* seropositivity rates. Seroprevalence of *T. gondii* infection in this pregnant population is similar to the only other report from Myanmar, where multiparity was also identified as a significant association. *Toxoplasma* infection is important in pregnant women. Nevertheless, in this marginalized population, this infection may be given less priority, due to resource constraints in providing the most basic components of safe motherhood programs.

*Toxoplasma gondii* parasitic infection is recognized as part of the group of CHEAP TORCHES (Chicken pox and shingles; Hepatitis C, D, and E; Enteroviruses; Acquired immunodeficiency syndrome [human immunodeficiency virus]; Parvovirus B19; Toxoplasmosis; Others [Group B *Streptococcus*, *Listeria*, *Candida*, Lyme disease]; Everything else sexually transmitted [*Neisseria gonorrheae*, *Chlamydia*, *Ureaplasma urealyticum*, human papillomavirus]; Syphilis) pathogens.^1^ These pathogens are classically considered as important etiological agents for perinatal and congenital infections.^2^ Late ocular and neurological manifestations have been reported in *T. gondii* congenital infections. Nevertheless, such complications can be found in other congenital infections.^3^ Other poor obstetric toxoplasmosis outcomes include spontaneous abortion, stillbirth, and preterm labor. Infections occurring early in pregnancy (first and second trimester) generally harm the fetus the most. Infection prior to pregnancy is not associated with poor outcomes.^3^^,^^4^

Prevalence rates in pregnant women in southeast Asia vary, with higher seroprevalence described for Indonesia (> 60%) and Malaysia (49%).^5^^,^^6^ Reported prevalence in Thailand is between 2.8% and 53.7%.^7^^–^^13^ Results from one study on pregnant women in Yangon, Myanmar, showed a seroprevalence of 30.7% (95% confidence interval [CI] = 27.9–37.2).^14^

Associated risk factors reported for *Toxoplasma* infection are older age, multiparity, drinking unclean water, contact with cats, lower educational levels, and less knowledge of toxoplasmosis.^9^^,^^10^^,^^14^ In one study, of 2,598 pregnant women in Thailand, Philippines, and Malaysia, only 11% had heard or read about toxoplasmosis.^15^ Screening for *T.* g*ondii* antibodies in pregnant women can identify women at risk for toxoplasmosis. In cases where IgG and IgM antibodies are detected, an IgG avidity assay can be used to rule out recent *Toxoplasma* infection.^16^

With this study, we aimed to give a better understanding on the seroprevalence of *T. gondii* infection in women from Myanmar living along the Thailand–Myanmar border. The Shoklo Malaria Research Unit (SMRU) is an operational field research unit providing humanitarian health care at three sites at the Thailand–Myanmar border and conducting research on pathogens of local importance. One of the clinics is located in the largest refugee camp for Myanmar refugees in Thailand (Maela refugee camp) and serves a population of around 45,000 people. The two other clinics, Maw Ker Thai clinic and Whang Pha clinic, provide health care for migrant workers in the area. Refugees and migrants are mainly of Karen ethnicity, coming from Karen State in Myanmar. SMRU medical team provides free antenatal care for pregnant women in the area and educates women in safe motherhood and prevention of infectious diseases in pregnancy.

This study was approved by the Oxford Tropical Research Ethics Committee (OXTREC: 28–09) and by the local committee with whom all research items were discussed before approval, the Tak Province Border Community Advisory Board (TCAB-4/1/2015). This was a retrospective analysis of stored serum leftover after completing the standard tests for first antenatal screen. A random selection of 200 samples (one excluded because samples were from the same woman) was chosen for analysis. This represented 3.4% (199/5,883) of all women who attended antenatal care clinics between January 2014 and July 2015. Serum samples were stored at −80°C. Data linkage between laboratory samples and baseline demographic data completed the set of variables for analysis.

Serum samples were tested using *T. gondii* enzyme-linked immunosorbent assay kits for the detection of IgG and IgM antibodies (Novatec, Dietzenbach Germany). Thresholds were set at 35 IU/mL and 10 Nephelometric Turbidity Units for positive IgG and IgM antibodies, respectively. Serum samples that were IgM antibody positive were tested for avidity using *T. gondii* IgG Avidity test (Novatec). Avidity results of 40% or higher reflected past infection.

Analysis of the data (Supplemental Table) was done using SPSS for Windows^™^ (version 20.0, SPSS Inc., Armonk, NY). Demographic data are presented as frequencies and percentages. Univariate analysis using the χ^2^ test was used to identify risk factors for *Toxoplasma* infection. The test was considered significant with *P* < 0.05. The odds ratios were calculated for categorical data. Poor obstetric outcome was defined as a history of miscarriage, stillbirth, neonatal death, or preterm labor in past pregnancies.

Overall, the seroprevalence of *Toxoplasma* infection was 31.7% (95% CI = 25.6–38.4). To our knowledge, there is only one other study in pregnant women from Myanmar, reporting similar results with a seroprevalence of 30.7%.^14^ Positive samples included three samples that were IgM positive, but avidity testing indicated that these were all past infections. Of the three sampling sites, ethnic background varied the most (Table 1

**Table 1:** Baseline characteristics of pregnant refugees and migrants enrolled to SMRU antenatal clinics

Characteristics	Total	Refugee	Migrant	
	*N* = 199	Maela	Maw Ker Thai	Wang Pha
		*N* = 78	*N* = 58	*N* = 63
Age in years, mean ± SD (range)	26 ± 7 (16–46)	26 ± 7 (17–46)	26 ± 9 (16–43)	26 ± 6 (17–43)
≥ 35 years of age, % (*n*)	16.6 (33)	14.1 (11)	25.9 (15)	11.1 (7)
Gravidity, median (range)	2 (1–12)	2 (1–12)	2 (1–7)	2 (1–9)
Parity, median (range)	1 (0–8)	1 (0–8)	1 (0–6)	1 (0–7)
Parity ≥ 3, % (*n*)	22.1 (44)	20.5 (16)	29.3 (17)	17.5 (11)
Primigravidae, % (*n*)	35.7 (71)	38.5 (30)	34.5 (20)	33.3 (21)
Poor obstetric history,* % (*n*)†	41.4 (53/128)	37.5 (18/48)	42.1 (16/38)	45.2 (19/42)
Literate (self-reported), % (*n*)	57.3 (114)	62.8 (49)	63.8 (37)	44.4 (28)
Soil-transmitted helminths in stool, % (*n*)‡	11.7 (23/197)	19.7‡ (15/76)	8.6 (5/58)	4.8 (3/63)
Months current address, median (range)	48 (1–468)	108 (1–468§)	12 (1–264)	24 (1–384)
At current address > 1 year, % (*n*)	66.8 (133)	89.7 (70)	48.3 (28)	55.6 (35)
Ethnic/religious group, % (*n*)				
Karen	60.8 (121)	83.3 (65)	36.2 (21)	55.6 (35)
Muslim	5.5 (11)	14.1 (11)	0	0
Burman	28.6 (57)	1.3 (1)	53.4 (31)	39.7 (25)
Other∥	5.0 (10)	1.3 (1)	10.3 (6)	4.8 (3)

ANC = antenatal care; SMRU = Shoklo Malaria Research Unit; SD = standard deviation.

* Poor obstetric outcome (miscarriage, stillbirth, neonatal death, or preterm labor in history).

† Primigravida excluded.

‡ Two missing.

§ Maela camp was established 33 years ago (in 1984) and a small proportion of women who attend SMRU ANC in Maela refugee camp give an address outside the camp which explains the 468 months (39 year) maximum value.

∥ Mon, Kachin, Pa Oh.

).

Maternal characteristics were compared according to *T. gondii* serostatus (Table 2

**Table 2:** Risk factors associated with toxoplasmosis serostatus

Characteristics	Seronegative	Seropositive	OR (95% CI)
	*N* = 136	*N* = 63	*P* value
≥ 35 years, % (*n*)	13.2 (18)	23.8 (15)	2.049 (0.955–4.393), *P* = 0.068
Parity ≥ 3, % (*n*)	16.9 (23)	33.3 (21)	**2.457 (1.233–4.895), *P* = 0.016**
Poor obstetric history,* % (*n*)†	40.2 (33/82)	43.5 (20/46)	1.142 (0.550–2.373), *P* = 0.852
Literate (self-reported), % (*n*)	56.6 (77)	58.7 (37)	1.090 (0.595–1.998), *P* = 0.878
Soil-transmitted helminths in stool, % (*n*)‡	7.1 (14)	4.6 (9)	1.468 (0.598–3.601), *P* = 0.474
Refugee, % (*n*)	39.0 (53)	39.7 (25)	0.971 (0.527–1.788), *P* = 1.000
At current address > 1 year, % (*n*)	64.0 (87)	73.0 (46)	1.524 (0.790–2.941), *P* = 0.258
Ethnic/religious group, % (*n*)			
Karen	71.1 (86)	28.9 (35)	OR not calculated, *P* = 0.259
Muslim	45.5 (5)	54.5 (6)	
Burman	64.9 (37)	35.1 (20)	
Other§	80.0 (8)	20.0 (2)	
Category Muslim compared with other groups, % (*n*)	3.7 (5)	9.5 (6)	2.758 (0.809–9.406), *P* = 0.106

CI = confidence interval; OR = odds ratio. Data are % (*n*). Bold font indicates *P* value < 0.05.

* Poor obstetric outcome (miscarriage, stillbirth, neonatal death, or preterm labor in history).

† Primigravida excluded.

‡ In order of commonality of detection: *Ascaris lumbricoides*, *Trichuris trichiura*, hookworm, *Strongyloides stercoralis*; data missing for two cases.

§ Mon, Kachin, Pa Oh.

). Only the proportion of women with parity of three or above had a higher seropositivity rate. Factors such as age of 35 years or above and being Muslim, were associated with higher proportion of *Toxoplasma*-seropositive status, albeit of no statistical significance (Table 2). The proportion of positive helminth or protozoa examination at the first antenatal visit and a poor past obstetric outcome (miscarriage, stillbirth, neonatal death, or preterm labor in history) in women with gravidity of one or above were not higher in women with *Toxoplasma*-seropositive status. In short, toxoplasmosis serostatus of the community here did not confer significant difference in their pregnancy outcomes (Table 3

**Table 3:** Pregnancy outcome by toxoplasmosis serostatus

Characteristics	Total *N*	Seronegative	Seropositive	OR (95% CI), *P* value
		*N* = 136	*N* = 63	
Lost to follow-up, % (*n*)	199	19.9 (27)	25.4 (16)	1.374 (0.678–2.786), *P* = 0.459
Miscarriage, % (*n*)	155	10.1 (11)	4.3 (2)	0.392 (0.083–1.842), *P* = 0.346
Singleton birth (*N* = 142)				
Stillborn, % (*n*)	142	2.1 (2)	0	OR not calculated, *P* = 1.000
Congenital abnormality, % (*n*)	142	3.09 (3)	0	OR not calculated, *P* = 0.552
Gestational age, mean ± SD (range), weeks (*n*)	137*	39.1 ± 1.5 (33.0–41.2) (*N* = 92)	38.6 ± 1.9 (30.6–41.1) (*N* = 45)	*P* = 0.126
Preterm delivery < 37 weeks, % (*n*)	137*	8.7 (8)	8.9 (4)	1.024 (0.291–3.601), *P* = 1.000
Birthweight mean ± SD (range), weeks (*n*)	129†	3,026 ± 452 (1,880–3,920) (*N* = 86)	2,892 ± 413 (1,470–3,590) (*N* = 43)	*P* = 0.096
SGA, % (*n*)	129†	19.8 (17)	18.6 (8)	*P* = 1.000
Apgar 5 minutes < 7, % (*n*)	125‡	2.44 (2)	0	*P* = 0.545

CI = confidence interval; OR = odds ratio; SGA = small for gestational age (< 10th percentile); SD = standard deviation.

* Only singleton pregnancies and live-born neonates without congenital abnormalities and estimated gestational age (EGA) > 28 weeks.

† Only singleton pregnancies and live-born neonates without congenital abnormalities and EGA > 28 weeks and birthweight only reported if measured in the first 72 hours of life.

‡ Three home deliveries and one birth at hospital and no known Apgar scores.

).

*Toxoplasma* seroprevalence varies widely worldwide with high prevalence rates described in Latin American countries (reports of > 70% in Brazil). In the southeast Asian region, higher prevalence rates are reported in Indonesia and Malaysia.^5^ Socioeconomic factors, environmental factors, religious beliefs, and hence lifestyle factors (such as pets of choice), can influence the population risk for *Toxoplasma* infection.^4^^,^^5^

In pregnant women, multiparity, older age, and a history of poor obstetric outcome have been previously described as risk factors resulting from exposure to *Toxoplasma*.^14^^,^^17^^–^^20^ In our study, only multiparity was significantly associated with *Toxoplasma* seropositivity. While there were trends for a higher proportion in women of age 35 years and above to carry *Toxoplasma*-seropositive status, the small sample size recruited for the study may preclude detection of other significant associations. There was a higher proportion of the Muslim population in the refugee camp that carried *Toxoplasma*-seropositive status. This is probably due to lifestyle or occupation-related transmission, as livestock animals (cattle, sheep, goat) are reared, slaughtered, and sold mostly by this group of population. Frequent contact with animals that can serve as *T. gondii* reservoirs coupled with inadequate hygiene practice, eating raw or undercooked meat, and drinking unclean water are previously reported as risk factors for higher *Toxoplasma* seroprevalence.^9^^,^^10^^,^^17^ However, lifestyle practices such as eating raw or undercooked meat or living with cats were not recorded during the study and therefore could not be taken into account for our data interpretation.

Health education programs, screening, and prevention are advocated by different studies to decrease the numbers of *T. gondii* infection in the pregnant population and to prevent adverse outcomes in neonates and later in life.^9^^,^^10^^,^^14^^,^^15^ Congenital toxoplasmosis manifests in the form of still birth, miscarriage, and progressive visual, audio, cognitive, and motor impairments to the affected children. Timely treatment improves outcomes and therefore careful monitoring and follow-up of mothers and children should be done if women are suspected for *Toxoplasma* infection in pregnancy. It would not be cost-effective to offer screening in this population of pregnant women. The high-risk group requires further refinement which could help identify those who could be considered for *Toxoplasma* infection screening during pregnancy. Nevertheless, in this and likely other marginalized populations, *Toxoplasma* parasitic infection may be given less priority, due to resource constraints in providing even the most basic components of safe motherhood programs. Reinforcing basic handwashing with soap and water, after working and before food preparation and eating, is likely to be the most useful, practical, and cost-effective health-care policy in this population.

## Supplementary Material

Supplemental Tables.
